# The Fly

**Published:** 1876-07

**Authors:** 


### THE FLY.

BY H.

"Fly"—def.—*musca domestica.* Here you have the "bird" as fully described as it is possible, and in only two words; translated into familiar English for common understanding, and what is more complete—*a domestic muss.*

His activity is wonderful—filled with a vague fancy to do forty things at once, he never settles to anything. Having a most extensive acquaintance he needs no preliminary forms of introduction, but proceeds to impose his presence upon you on occasions of his own selection. He settles upon the first accessible spot offering him foot-hold and proceeds to make a running account of your personal effects. Does your bald head shine with unusual smoothness, his little eye sees in it an inviting parade ground, and he races with utmost speed across its barrenness. Your sleeping face excites within him a desire for inspection. He knows you will be highly tickled with the idea, hence he does not delay the execution of his plan. He stops at your nostrils merely long enough to tempt you to try to overtake him in an open-handed way—laughs to know that he was not there when you was, and gets your ear in spite of you.

To the moralist he affords unending reflections. No utilitarian views ever enter into his calculations, hence no estimate of lost forces expended yearly by the great masses of man and animal-kind in meeting the wear of physical powers expended in brushing and fighting the "bird." One swing of a cow's tail must waste some amount of force, this repeated by the number per hour, per day, and per month is incalculable. Some statistician should estimate how much horse power is thus wasted in gratifying the humor of one subject. 'Twould be interesting and instructive. Here is motive power enough to run all the machine shops of the country. How vast could be the saving of food consumed to support this particular demand for it.

Innocent as a fly! How often shall this be said to remind the little ones of our households not to exercise cruelty to their helpless bodies when finally cornered and caught in the angle of some unfriendly window-sash? Ignorant as a fly would be better. Who ever saw an educated fly—one that had an appeciable amount, sufficient to distinguish the right from the wrong (side of a milk pitcher)? Every misfortune of the fly, every disagreeable and annoying feat it performs, is the result of its ignorance and folly. It is cursed by nature—is born grown up, all flies are. They come to us the first day of May in full size and health, prepared to commence business of their kind. All flies are the same size, and there are no young or old. A fly has no childhood, no experience, no parental education—'tis a thing created thus a fool. It does not know what to do, hence is always wrong. Every misfortune, every disagreeable feat it does comes from its folly. It don't know the distance from the cream-pitcher's edge to the cream below. It slops in all untaught to die in the cream of life, unless raised by an unkind hand that wished it dead before it had made the attempt to investigate what it had no brains to comprehend. Our dishes, during this *tempus fugit,* are the receptacle of many of these our estee(a)med friends, as they would have themselves called. The house-wife kneads them into the bread, and the good man, with smiles on his parting lips, smears the fly-caught butter over the remains, unconscious of the fly at the time. With what grace and benedictions the good wife wafts them outward the open door, upon breezes of a flitting mantle or other garment. We see them go and bless them, as they turn for a moment to look at us from the other side of the netted screens. In their loss to us we still perceive some new gain and are consoled. Debarred from getting to us, our blessings go with them as they betake themselves to the next door neighbor, who will receive them with open doors.

Some day or summer when the great eternal fitness of everything is revealed to us, the musca domestica shall go forth with encomiums from us all. Till then, no blame should be to those who prepare the numerous patent fly-traps. These geniuses are to be encouraged. For you, flies, we pray that food to please and occupy your spare time may abound with death, and all shall be well with thee, muscæ. For flies are an abomination in our sight. By quarts of dead ones we would know thee.

We are told that nothing has been created in vain, and that these flies are great scavengers in the ecomomy of nature. If so, why don't they keep to their work according to their contract?

Do we suppose that the fresh made pies, puddings and pantry sauces are so full of utter rottenness that all the flies in the room must be hurriedly congregated, to stand in lines around the edges of the dishes containing it, with their noses embeded in its sweetness? We are competent to manage all this scavenger business for ourselves. Out with them to the stables and by places. They wont bother any one there, nor will any one bother them, even if they want to carry the whole of the garbage away and dump it into the river or elsewhere. Go to, now, yes, go all of you to this useful field of labor. Get to business—get up rings in your own appointed line and make yourselves useful. Don't buzz us any more!

